# PIK3CA mutation–induced immune microenvironment remodeling sensitizes cervical cancer to immunotherapy

**DOI:** 10.3389/fimmu.2026.1780752

**Published:** 2026-03-25

**Authors:** Fei Zhu, Xingyun Xie, Cong Wang, Jin Liu, Ying Chen, Dan Hu, Qin Xu

**Affiliations:** 1 Departments of Gynecology, Clinical Oncology School of Fujian Medical University, Fujian Cancer Hospital, Fuzhou, China; 2 Department of Gynecologic Oncology, Shandong Cancer Hospital and Institute, Shandong First Medical University and Shandong Academy of Medical Sciences, Jinan, China; 3 Department of Pathology, Clinical Oncology School of Fujian Medical University, Fujian Cancer Hospital, Fuzhou, China

**Keywords:** cervical cancer, immunotherapy, PIK3CA mutation, single-cell RNA sequencing, tumor microenvironment

## Abstract

PIK3CA is one of the most frequently mutated genes in cervical cancer (CC). However, its clinical utility is hampered by paradoxical treatment-dependent outcomes, restricting its application in precision oncology. To address this issue, we constructed a high-resolution single-cell transcriptomic atlas of the CC tumor microenvironment. It was found that PIK3CA mutations induce a dichotomous TME, simultaneously associated with marked T-cell inflammation and resistance to adaptive immune responses. Malignant epithelial subsets induce CD8^+^ T-cell exhaustion through both canonical PD-L1-PD-1 signaling and the non-canonical SPP1-CD44 axis. Additionally, PIK3CA mutations enrich for MMP9^+^ macrophages that promote tumor angiogenesis through ANGPTL4 signaling. This dual landscape of T-cell exhaustion and active angiogenesis provides a framework for the observed synergy between PD-1 blockade and anti-angiogenic therapies. The findings demonstrate that the presence of PIK3CA mutations is a key predictive biomarker for guiding combination immunotherapy in CC and identify a rational basis for co-targeting distinct immune and vascular resistance pathways.

## Introduction

Immunotherapy now forms part of standard first-line and second-line treatment regimens for patients with cervical cancer (CC). This application of immunotherapy has substantially improved patient prognosis, particularly for those with advanced recurrent and metastatic CC, where the combination of immunotherapy with anti-angiogenic therapy has increased the overall response rate (ORR) from less than 20% to over 50% (1). Traditional indications for immunotherapy have relied primarily on the PD-L1 Combined Positive Score (CPS)and the tumor mutation burden (TMB) (2, 3); however, a significant proportion of patients remain unresponsive to treatment (4, 5). Therefore, the identification and refinement of biomarkers that can accurately predict responses to immune checkpoint blockade (ICB) is important for optimizing treatment strategies.

Genomic analyses from multiple clinical cohorts have consistently identified PIK3CA as the most frequently mutated gene in CC (6–8). The PI3K/AKT/mTOR pathway, which includes the PIK3CA protein, mediates essential processes involved in the early stages of cancer onset and progression, including modulation of the cell cycle and tumor cell growth, metabolism, proliferation, and survival (9). Genetic alterations resulting in hyperactivation of the PI3K/AKT/mTOR pathway are observed in numerous cancer types (10–13). The high frequency of PIK3CA mutations in CC suggests their complex involvement in tumorigenesis and response to therapy, with their prognostic significance exhibiting a striking treatment-dependent paradox. In surgically treated patients, PIK3CA mutations are associated with improved relapse-free survival (14), whereas in concurrent chemoradiotherapy (CCRT)-treated cohorts, they are correlated with significantly poorer overall survival and cancer-specific survival (15, 16). This paradoxical effect is thought to result from differences in therapeutic pressures: while surgical resection removes the primary tumor, potentially mitigating the impact of PI3K/AKT-driven resistance, sustained pathway activation during CCRT may instead promote therapeutic failure. Notably, despite the demonstrated involvement of EGFR, its targeting in combination with CCRT failed to improve DFS, with PI3K pathway-mutated tumors exhibiting complete resistance (0/14 vs. 14/52 response rates) and diminished survival trends (16).

PIK3CA mutations have emerged as critical biomarkers in precision oncology, for instance, in breast cancer, where they are apparent in approximately 40% of HER2-negative advanced breast cancer (17–19). Phase 3 trials have also demonstrated that PI3Kα-selective inhibitors can significantly extend both progression-free and overall survival in patients harboring PIK3CA-mutated tumors, suggesting the potential of PIK3CA as a target for personalized therapy (19, 20). Our previous clinical study, supported by independent investigations, showed that PIK3CA-mutated CC tumors displayed enhanced responsiveness to combined treatment with PD-1 inhibitors and anti-angiogenic therapy, suggesting their potential as predictive markers for precision immunotherapy (21–24). Mechanistically, it was hypothesized that PIK3CA mutations reshape the tumor microenvironment (TME) into a state more conducive to immune recognition, although the specific cellular and molecular mechanisms driving this process remain largely unknown. Given the complex heterogeneity of the TME, high-resolution analytical approaches are necessary to unravel these underlying dynamics. Single-cell RNA sequencing (scRNA-seq) can address this challenge by enabling detailed profiling of gene expression at the individual cell level, allowing elucidation of intricate interactions among the diverse cellular components of the TME (25).

Here, scRNA-seq was used to dissect the TME of PIK3CA-mutated and wild-type CC tumors to elucidate the mechanisms by which these mutations induce sensitivity to immunotherapy. The findings revealed that PIK3CA mutations orchestrated a dichotomous tumor microenvironment, in which they promote a state of immune heterogeneity that renders tumors responsive to immunotherapy, while also inducing angiogenesis, thus providing a mechanistic basis for the observed clinical synergy with anti-angiogenic agents. These insights position PIK3CA mutations as a pivotal predictive biomarker for stratifying CC patients toward optimized combination regimens, and provide potential therapeutic targets.

## Results

### Single-cell transcriptomic atlas of PIK3CA-mutant and wild-type cervical cancer

To first establish the clinical and biological relevance of PIK3CA mutations in CC, a comprehensive analysis of The Cancer Genome Atlas (TCGA) genomic dataset was conducted. The results confirmed that PIK3CA was one of the most frequently mutated genes in the CC cohort (**Supplementary Figure 1A**). This finding aligns with the mutation profiles observed in our previous clinical study as well as the results from earlier studies (6–8, 21, 23). A pairwise interaction analysis revealed a significant co-occurrence between PIK3CA mutations and alterations in several other genes, including NEB, ADGRV1, USH2A, and SYNE1, suggesting potential cooperative roles in tumorigenesis (**Supplementary Figure 1B**). Critically, OncodriveCLUST analysis identified significant positional clustering of PIK3CA mutations, confirming that they are under positive selection and function as drivers in CC (**Supplementary Figure 1C**).

After the establishment of PIK3CA as a key driver, its influence on the TME was investigated using bulk RNA-seq data from TCGA. The results of single-sample gene set enrichment analysis (ssGSEA) suggested that the effects were both complex and contradictory. PIK3CA-mutant tumors showed significantly higher infiltration of CD56dim NK cells, a cytotoxic subset known for potent anti-tumor activity. Conversely, wild-type (WT) tumors were characterized by a greater abundance of macrophage signatures (**Supplementary Figure 1D**). These results suggested that the TME of mutant tumors was more cytotoxic, but did not explain the full context of immune cell interactions. The inherent limitations of bulk transcriptomics, which average signals from all cell types, do not permit an understanding of the complete immune landscape and how these distinct cell populations are orchestrated. This necessitated the use of a higher-resolution approach.

**Figure 1:**
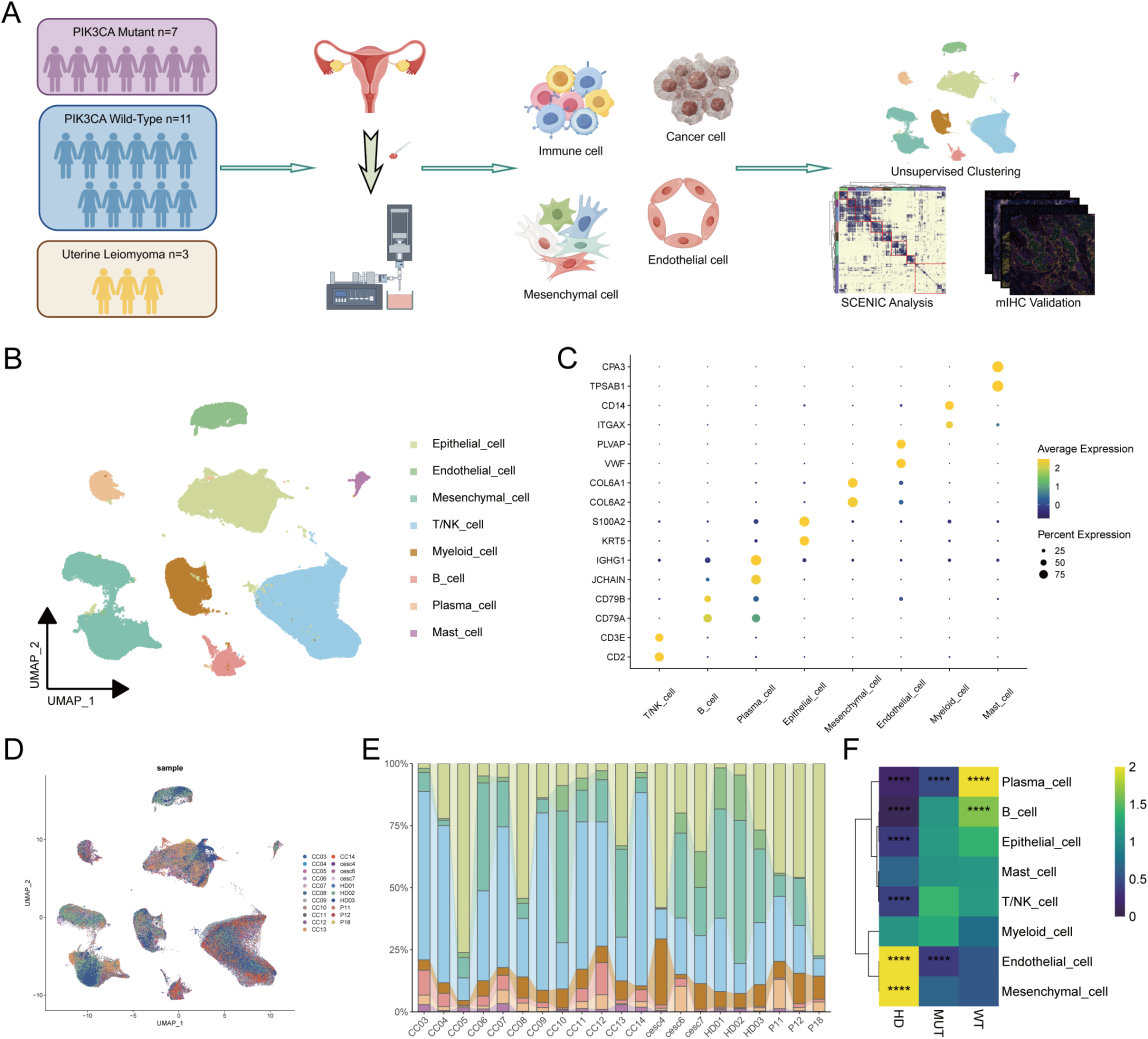
**(A)** The sample collection, sequencing, and analysis pipeline used in this study. **(B)** Uniform Manifold Approximation and Projection (UMAP) plot showing eight annotated subclusters following dimensionality reduction and clustering. **(C)** Dot plots showing the expression patterns of specific marker genes used for annotation of the eight subclusters, with two representative genes selected for each cluster. The proportions of cells with positive expression are indicated by dot size, and expression levels are represented by color intensity. **(D)** UMAP plot demonstrating batch-associated variation among the 21 analyzed samples. **(E)** Proportional representation of the eight subclusters in the different samples, with colors corresponding to those in **Figure 1A**. **(F)** Heatmaps showing the odds ratios (ORs) of major subclusters across different groups. Statistical significance is indicated by asterisks, with “****” representing p < 0.0001 and either OR > 1.5 or OR < 0.5.

To deconstruct the complexity of immune interactions at the single-cell level, a comprehensive scRNA-seq cohort was established. This included a total of 21 samples, specifically, 7 PIK3CA-mutant (MUT) tumors, 11 WT tumors, and 3 uterine leiomyoma (UL) samples as reference (**Figure 1A**; **Supplementary Table 1**). This meta-cohort integrated three newly sequenced samples with publicly available datasets from Liu and Fan (26, 27). After stringent quality control, a total of 173,572 high-quality cells were retained for downstream analysis. Unsupervised clustering and annotation based on canonical marker genes revealed eight major cell lineages, comprising Epithelial, Endothelial, Stromal, T/NK, B, Plasma, Mast and Myeloid cells, all of which were present in all samples, albeit in varying proportions (**Figures 1B–E**; **Supplementary Figures 1E, F**).

The single-cell analysis provided a higher-resolution perspective, building upon the bulk data observations. Analysis of differential cellular abundance revealed a marked remodeling of the cellular landscape in CC tumors, characterized by lower levels of endothelial and myeloid cells compared to healthy samples, as well as greater numbers of B and plasma cells in the MUT group relative to the WT group (**Figure 1F**). This intricate cellular reorganization lays the foundation for a deeper exploration of the functional states and interactions among these critical cell populations.

### Differences in the distribution of malignant epithelial subpopulations

**Figure 2:**
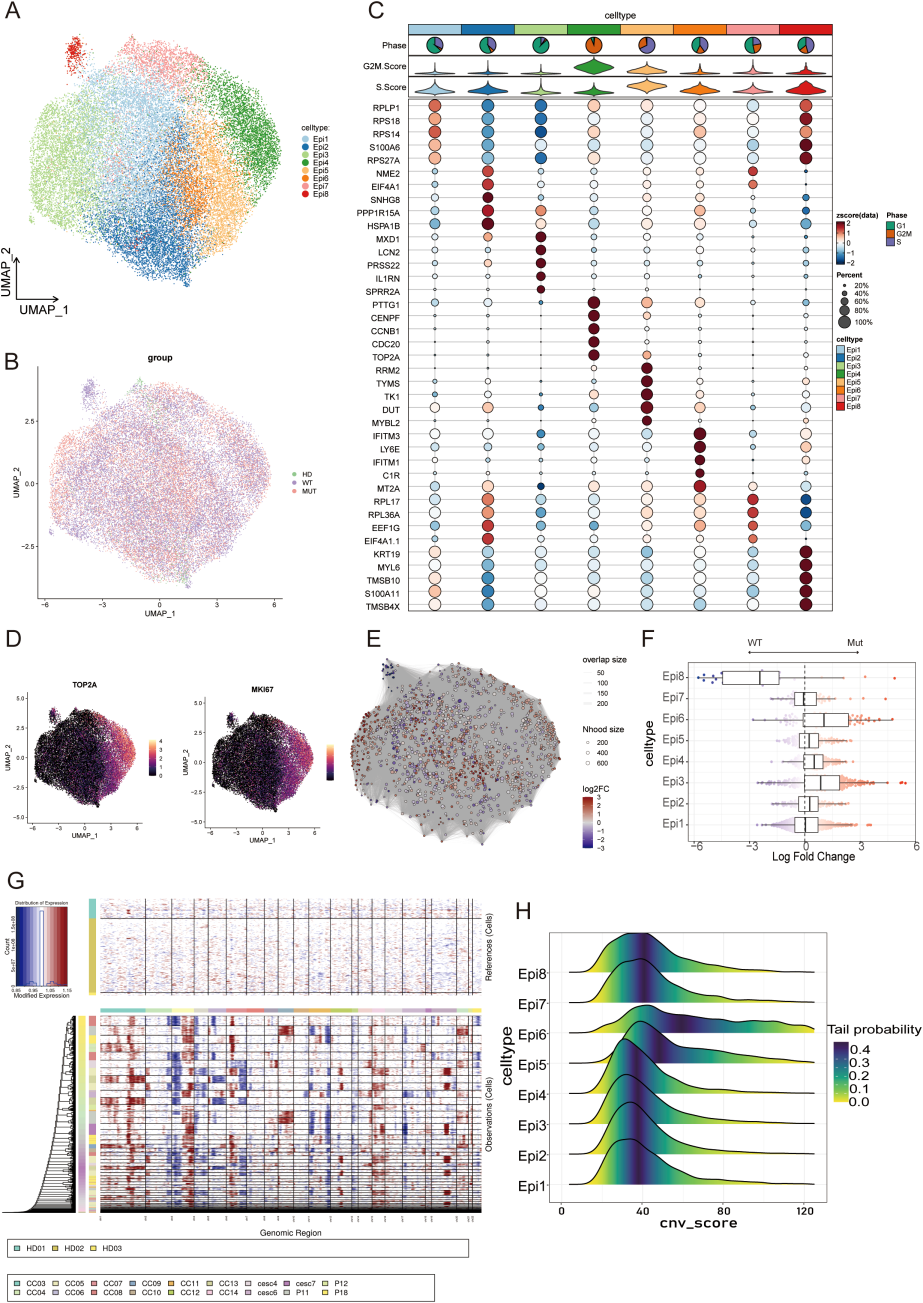
**(A)** UMAP plot showing the clustering of epithelial subpopulations into eight distinct subclusters. **(B)** UMAP plot showing epithelial cells from the HD, WT, and MUT groups, colored by sample identity to assess potential batch effects across groups. **(C)** The top layer of the figure includes pie charts and violin plots showing the proportions of cells in different cell-cycle phases within each subpopulation. The lower section shows dot plots of the top five most highly expressed genes in different epithelial subpopulations. Dot size represents the proportion of cells expressing each gene, and color intensity indicates the expression level. **(D)** UMAP plot showing the expression of TOP2A and MKI67. **(E)** Neighborhood graph from the Milo analysis comparing epithelial cells from MUT vs. WT samples. Each node represents a single neighborhood. Nodes are colored by the log2 fold change (log2FC) in abundance. Neighborhoods significantly enriched in the MUT group are colored red, while those enriched in the WT group are colored blue. **(F)** Beeswarm plots overlaid with box plots showing the distribution of neighborhood log2FC values across different epithelial cell subtypes. Colors correspond to the log2FC as in **(E)**. Box plots show the median (center line), interquartile range (IQR; box limits), and whiskers extending to 1.5x the IQR. **(G)** Copy number variation (CNV) landscape of tumor epithelial cells from the MUT and WT groups inferred using normal cervical tissue as reference. Red and blue regions represent chromosomal amplifications and deletions, respectively. **(H)** Ridge plot illustrating the distribution of inferred copy number variation (CNV) scores across epithelial cell subpopulations. CNV scores were calculated from the InferCNV analysis, with higher scores indicating greater chromosomal instability. Each ridge represents the density distribution of CNV scores for cells within a given cluster.

Subpopulation clustering of 31,756 epithelial cells was conducted to explore the differences among malignant cells. This analysis identified eight distinct epithelial subpopulations, each characterized by a unique gene expression signature (**Figures 2A, B**). Several conserved epithelial cell categories were identified, based on previous classifications, namely, subpopulation 1 with high expression of ribosomal genes, subpopulation 2 expressing genes related to the stress response, and subpopulation 4 associated with the cell cycle (26, 28). Evaluation of the cell cycle score for each epithelial subpopulation showed that subpopulation 4 had the highest G2M phase score (**Figure 2C**), indicating greater proliferative potential. Additionally, when proliferation-specific genes were scored using AUCcell, TOP2A and MKI67 had the highest scores in subpopulation 4 (**Figure 2D**). Subpopulation 6 was enriched with interferon genes, suggesting a significant role in immune regulation.

The miloR algorithm was used to assess epithelial cell-specific subpopulations among the groups. The results showed that Epi3 (logFc = 0.527) and Epi6 (logFc = 0.740) were higher in the MUT group, while the Epi8 subpopulation was more prevalent in the WT group (**Figures 2E, F**). Notably, the proliferative subpopulation 4 was more abundant in the MUT group, suggesting that PIK3CA mutations may confer a proliferative advantage to epithelial cells. Tumor cells with PIK3CA mutations often exhibit a higher degree of malignancy due to increased DNA copy number segment amplifications and deletions. The CNV status of each subpopulation was then evaluated using Infercnv, finding that subpopulation 6 exhibited a higher CNV score. This may explain the stronger interferon response observed in this subpopulation (**Figure 2G, H**).

### Functional and metabolic reprogramming driven by PIK3CA mutations

**Figure 3:**
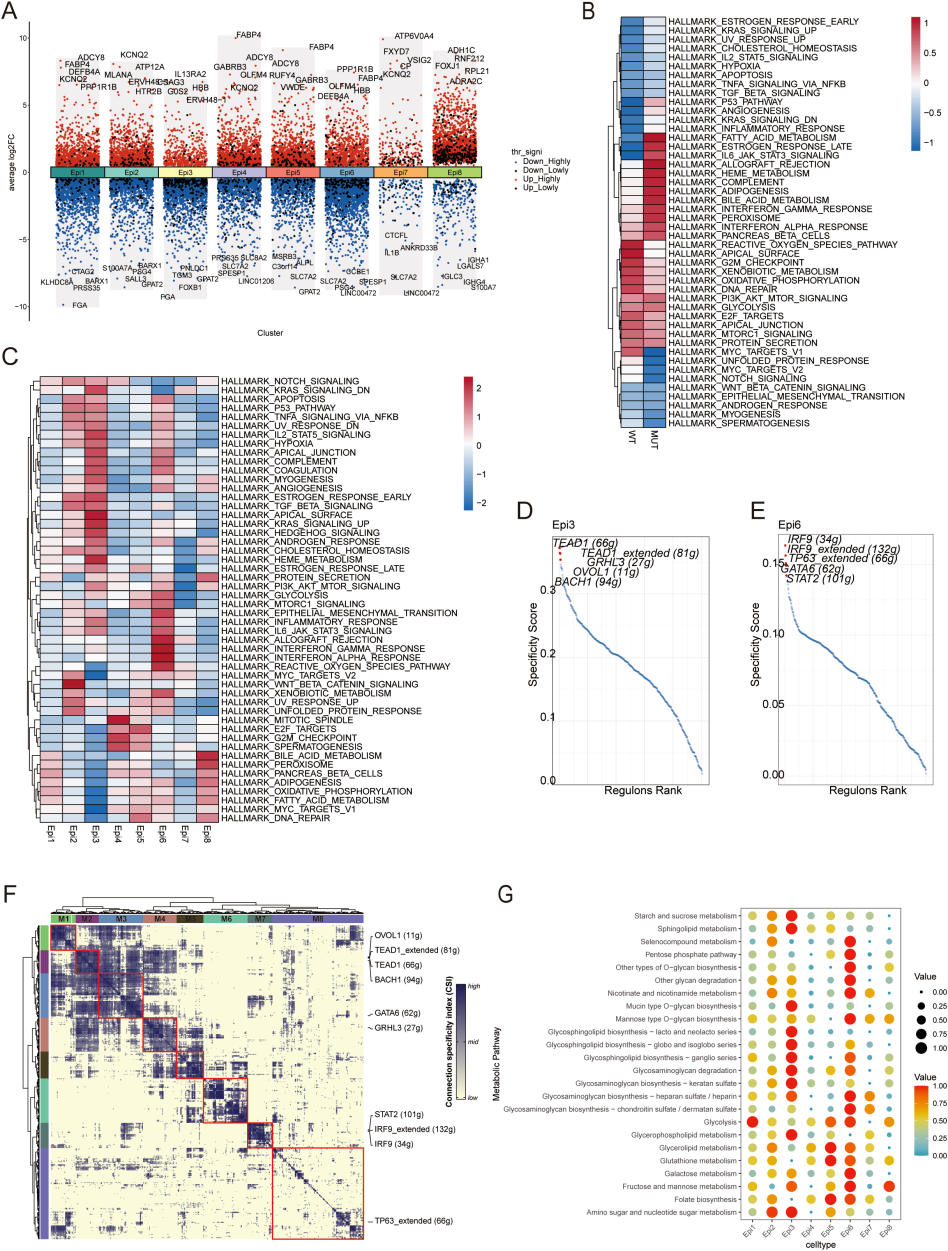
**(A)** Differential gene expression between the mutant and wild-type groups in eight epithelial subclusters. Upregulated and downregulated genes are displayed for each subcluster. Genes with adjusted p < 0.01 are highlighted in red; all others are shown in black. **(B)** Hallmark pathway activity scores for the mutant and wild-type groups, calculated via the AddModuleScore. **(C)** Hallmark pathway activity scores in the eight epithelial clusters, calculated via the AddModuleScore. **(D)** Rank for regulons in epithelial subclusters Epi3 based on the RSS. **(E)** Rank for regulons in epithelial subclusters Epi6 based on the RSS. **(F)** Identified regulon modules based on the regulon CSI matrix. **(G)** Dot plot showing the metabolic activity analysis of the eight epithelial subclusters using scMetabolism.

To assess the functional impact of PIK3CA mutations, the transcriptomic profiles of epithelial cells in MUT and WT tumors were compared. This showed that fatty acid-binding protein (FABP) genes were significantly upregulated in multiple epithelial subsets in the MUT group (**Figure 3A**), suggesting a broad reprogramming of lipid metabolism. This is consistent with the known role of PIK3CA mutations in promoting increased fatty acid uptake and utilization to support the elevated energetic and biosynthetic demands of rapidly proliferating tumor cells (29). Further pathway analysis revealed that MUT tumors were significantly enriched in interferon-gamma (IFN-γ), interferon-alpha (IFN-α), and IL-6/JAK/STAT3 signaling signatures (**Figure 3B**). Together, these initial findings indicate that PIK3CA mutations induce a complex phenotype characterized by both profound metabolic changes and a pro-inflammatory, “anti-viral-like” state.

For further deconstruction of this heterogeneity, the specific functional roles of the MUT-enriched subpopulations, Epi3 and Epi6, were investigated. This indicated that the two subsets had distinct, although complementary, pro-tumorigenic identities.

The Epi3 subpopulation appeared to be a hub for tumor progression, characterized by the co-enrichment of multiple potent signaling pathways (**Figure 3C**). It displayed the highest level of TGF-β signaling, a canonical inducer of the epithelial-mesenchymal transition (EMT), as well as high levels of angiogenesis, hypoxia, and Notch signaling. This combination suggests a cell state with enhanced invasive capabilities and a capacity to build its own blood supply, as well as adaptability to harsh microenvironments and the acquisition of cancer stem cell-like properties. Furthermore, strong activation of the TNF-α/NF-κB pathway suggested a state of chronic inflammation, potentially promoting cell survival and mediating immune evasion. SCENIC analysis identified the master transcription factors involved in this aggressive phenotype. The pro-metastatic regulon BACH1 and the core Hippo pathway effector TEAD1 were among the most highly active factors in Epi3 (**Figure 3D**). Further analysis of the entire regulon network revealed that these transcription factors belonged to a co-regulated module (Module 2, M2) that was specific to the Epi3 subpopulation (**Figure 3F**). Metabolically, Epi3 was enriched in pathways associated with the biosynthesis of glycosphingolipids and glycosaminoglycans (**Figure 3G**), suggesting it remodels its cell surface to directly suppress immune recognition by altering ligand-receptor interactions. Collectively, these features indicate that Epi3 may drive malignant progression.

In contrast, the Epi6 subpopulation was the primary source of the global inflammatory signals. It exhibited the highest activity scores for both the IFN-α/γ response and IL-6/JAK/STAT3 signaling (**Figure 3C**). This “anti-viral-like” signature represents a classic double-edged sword in tumor immunity. On one hand, a strong IFN signature is often a prerequisite for successful anti-PD-1/PD-L1 immunotherapy, as it indicates an inflamed “hot” TME where T cells are actively engaging the tumor. On the other hand, this same IFN signaling is a primary driver of adaptive immune resistance, a process where tumor cells upregulate immunosuppressive ligands such as PD-L1 to shut down the T cells attacking them (30, 31).

This dual capacity implies that Epi6 may be an overall regulator of TME inflammation. While its IFN signature could initially attract anti-tumor immune cells, the concurrent IL-6 signaling can lead to a separate immunosuppressive milieu, promoting T-cell exhaustion and the recruitment of myeloid-derived suppressor cells (MDSCs) (32, 33). The underlying transcriptional program for the IFN response was driven by strong activation of the IRF9 and STAT2 regulons, core components of the ISGF3 transcription complex in type I interferon signaling (**Figure 3E**). This links the observed phenotype directly to the canonical JAK/STAT pathway known to control PD-L1 expression. As in Epi3, these key regulons formed part of a distinct, co-regulated network (Module 7, M7) that was predominantly active in Epi6 (**Figure 3F**). Results suggested that Epi6 underwent specific metabolic reprogramming to support this high-energy signaling state and cope with the associated oxidative stress, seen in its significant enrichment in glutathione metabolism, the pentose phosphate pathway, and niacin/nicotinamide metabolism (**Figure 3G**), enhancing its antioxidant capacity and the generation of crucial cofactors such as NAD+/NADPH.

Taken together, the results indicate that PIK3CA mutations promoted a complex ecosystem of malignant cells, initiated by a broad shift toward lipid metabolism. This environment supported the emergence of functionally specialized subpopulations, with the Epi3 subset acting as the “driver” of physical invasion and immune suppression, controlled by the M2 regulon module, while the Epi6 subset functioned as the “signaler,” inducing an inflammatory milieu through the M7 regulon module. This functional synergy provides the basis for tumor progression and immune escape.

### PIK3CA mutations remodel the T/NK cell landscape toward an exhausted phenotype

**Figure 4:**
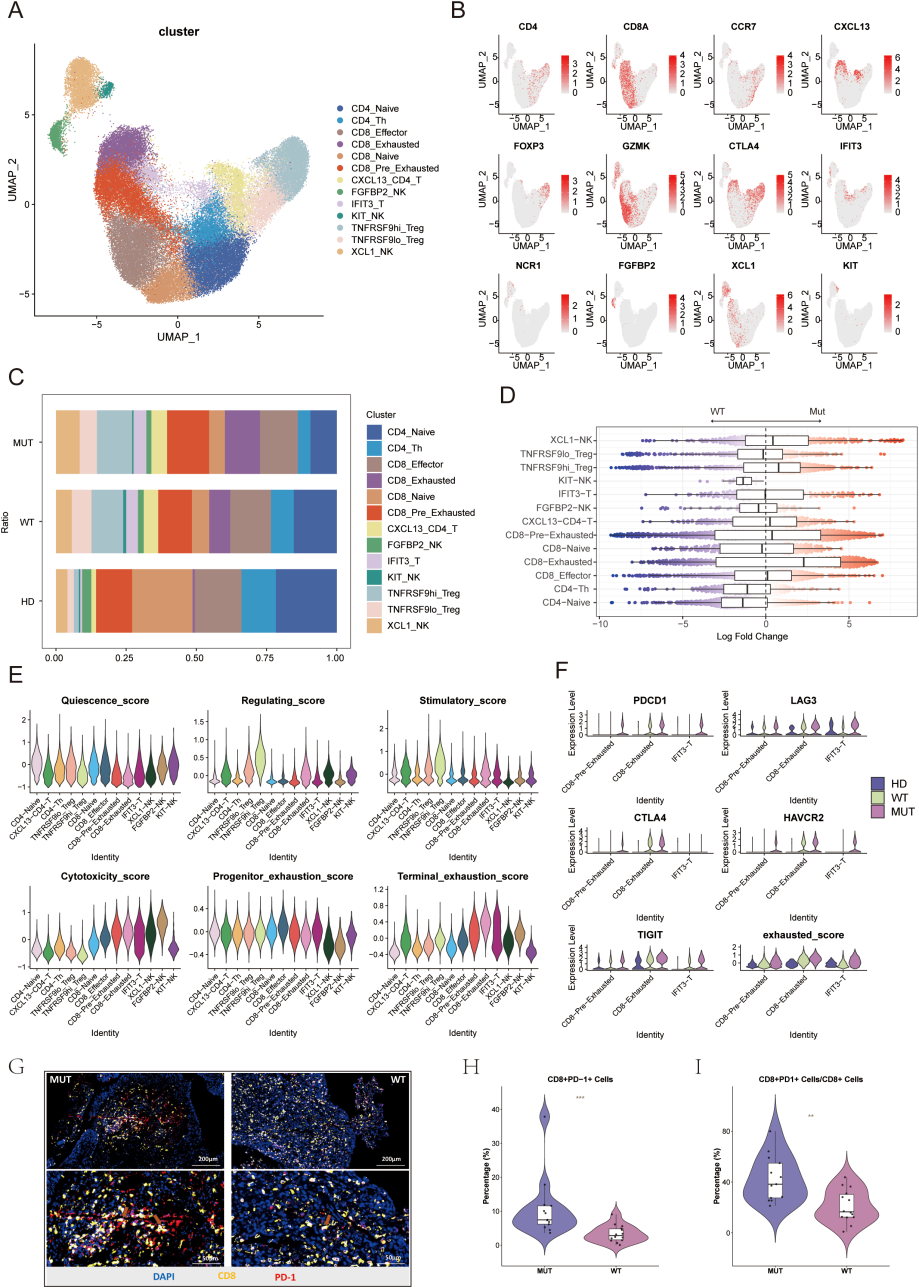
**(A)** UMAP plot showing the clustering and annotation of NK/T cell populations into 13 distinct subclusters. **(B)** UMAP plots showing marker gene expression for cell type identification. The legend indicates a color gradient of normalized expression. **(C)** Bar plots showing subcluster distributions in the UL, MUT, and WT groups. **(D)** Beeswarm plots overlaid with box plots showing the distribution of neighborhood log2FC values in different NK/T cell subtypes. Colors correspond to the log2FC as in 2E. Box plots display the median (center line), interquartile range (IQR; box limits), and whiskers extending to 1.5× the IQR. **(E)** Violin plots illustrating the distribution of module scores for NK/T subclusters, calculated based on predefined gene signatures. **(F)** Violin plots illustrating intergroup differences in the expression of exhaustion-related molecules and exhaustion gene signature scores in the three exhausted subclusters. **(G)** Representative mIHC staining of CD8^+^ PD1^+^ T cells in the MUT and WT groups. **(H)** Violin plot showing the proportion of CD8^+^ PD1^+^ T cells in the MUT and WT groups based on mIHC staining results. Each dot on the violin plot represents an individual sample,***p < 0.001. **(I)** Violin plot showing the proportion of CD8^+^ PD1^+^ T cells among total CD8^+^ T cells in the MUT and WT groups based on mIHC staining results. Each dot on the violin plot represents an individual sample, **p < 0.01.

High-resolution clustering of T and NK cells was then conducted to dissect the adaptive immune compartment, identifying 13 distinct subsets based on their transcriptional profiles and expression of canonical marker genes (**Figures 4A, B**; **Supplementary Figure 2A, B**). A broad comparison of subset proportions revealed a generally immunosuppressive TME compared to that of healthy tissue, characterized by a significant depletion of naive T cells (CD4_Naive, CD8_Naive) and a marked enrichment of regulatory T cells (TNFRSF9hi_Treg, TNFRSF9lo_Treg) (**Figure 4C**). This limited T-cell replenishment and presence of dominant regulatory populations are conducive to immune evasion.

How PIK3CA mutation status shapes this immune environment was then investigated. Analysis of differential abundance using MiloR, which compares cell neighborhood distributions, revealed significant differences between MUT and WT tumors. The CD8_Exhausted subset was significantly and predominantly enriched in the MUT group (logFC = 2.28). While not reaching statistical significance, the CD8_Pre_Exhausted subset also showed a clear trend of higher abundance in MUT tumors (**Figure 4D**). This progenitor-exhausted population is considered a key reservoir for the reinvigoration of anti-tumor immunity in response to checkpoint blockade (34, 35). Their increased presence, together with the terminally exhausted cells, suggests that the MUT TME is characterized by a state of chronic T-cell stimulation and subsequent dysfunction.

This exhausted state was then quantified by the calculation of exhaustion scores for all subsets, confirming that CD8_Exhausted, CD8_Pre_Exhausted, and a unique interferon-stimulated T cell (IST) subset (IFIT3_T) were the three most exhausted populations (**Figure 4E**). A direct comparison of these three subsets between the MUT and WT groups showed that expression of key exhaustion-related genes (PDCD1, LAG3, CTLA4, HAVCR2) and the overall exhaustion signature score were consistently higher in the MUT group (**Figure 4F**). Further multiplex immunohistochemistry (mIHC) staining verified the significantly higher abundance of CD8^+^ PD-1^+^ T cells in the MUT group compared to the WT group (**Figures 4G–I**). This finding confirms the earlier observation of a potent IFN signature in the malignant Epi6 subpopulation, suggesting a model where epithelial cell-derived interferons drive T-cell activation and subsequent exhaustion, a hallmark of adaptive immune resistance.

In contrast, the immune landscape of WT tumors appeared to rely more on cytotoxic NK cell activity. A cytotoxic FGFBP2_NK (CD56dim) subset, significantly more abundant in the WT group, was identified (**Figure 4D**). This finding, supported by the external TCGA data and survival analyses (**Supplementary Figures 2C, D**), suggests that an active NK cell response is a prominent feature of anti-tumor immunity in PIK3CA WT tumors.

In summary, PIK3CA mutant status delineates two distinct immune escape trajectories. Mutant tumors are characterized by a T-cell-inflamed but exhausted TME, providing a strong rationale for therapies targeting T-cell exhaustion. Conversely, WT tumors appear to suppress the cytotoxic NK cell response more effectively.

### Mutant epithelial cells drive the progressive exhaustion of CD8^+^ T cells via SPP1 and PD-L1 signaling

**Figure 5:**
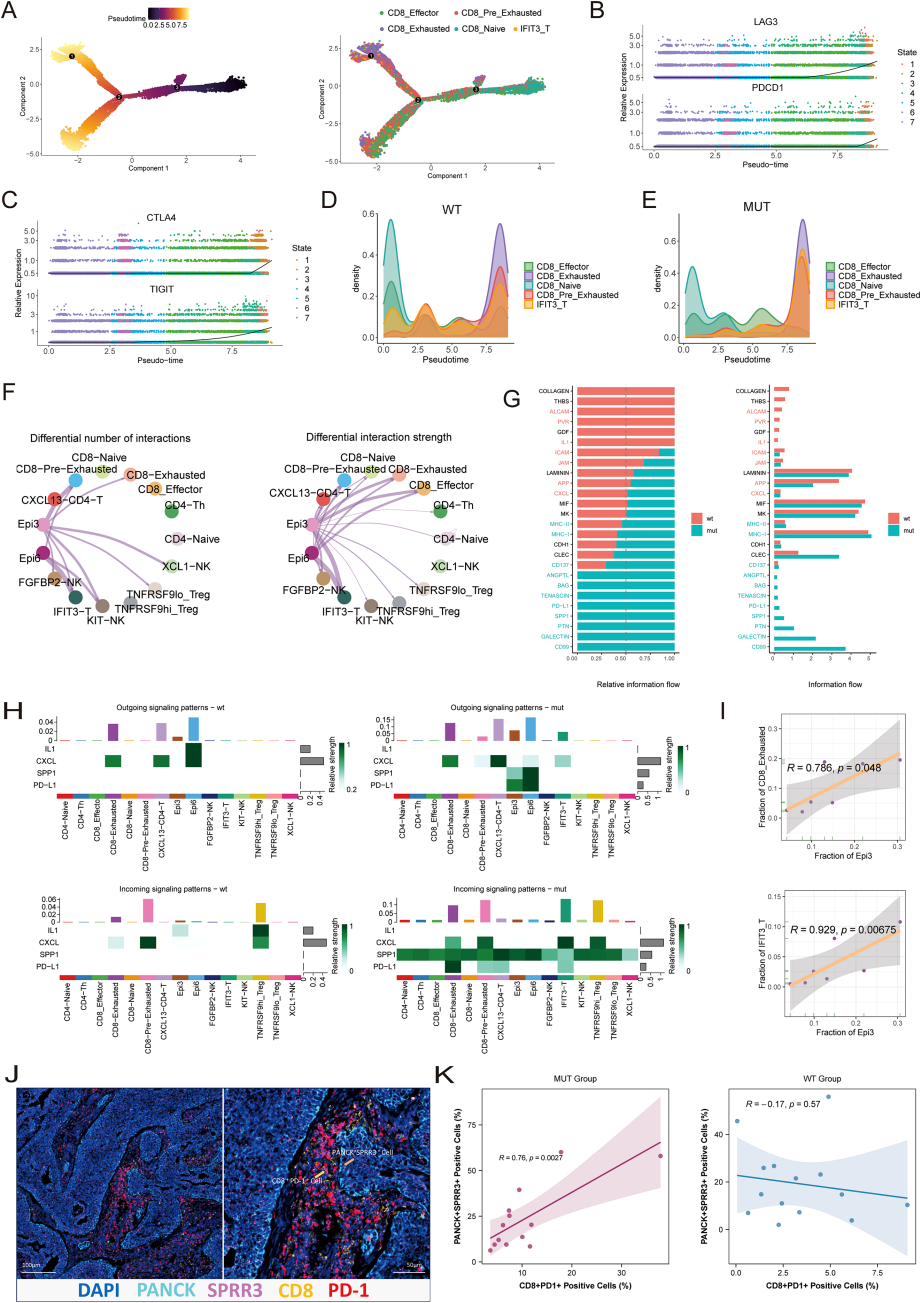
**(A)**. Monocle pseudotime trajectory analysis of CD8^+^ T cells. The left panel shows the inferred direction of pseudotime differentiation, indicated by a continuous color gradient. The right panel displays individual cells as dots colored by subcluster identity. **(B, C)**. Dynamic expression of exhaustion-related molecules along the CD8^+^ T cell trajectory. **(D, E)**. Ridge plots showing the distribution of different subclusters from the MUT and WT groups along the developmental trajectory of CD8^+^ T cells. **(F)** Circle plots illustrating cell-cell communication between Epi3/Epi6 and different NK/T cell subpopulations. The left plot shows the number of interactions; the right plot shows the interaction strength. **(G)**. Bar plots showing the relative proportion of cell-cell communication signals between epithelial cells and NK/T subpopulations (left) and the relative signal strength (right). **(H)**. Outgoing and incoming signals of four key communications in the MUT and WT groups. **(I)**. Scatter plots showing the positive correlation between Epi3 abundance and that of CD8_Exhausted cells (upper panel, Pearson’s correlation, R = 0.786) and IFIT3_T cells (lower panel, Pearson’s correlation, R = 0.929) in the MUT group. **(J)**. Representative mIHC staining showing colocalization between CD8^+^ PD1^+^ T cells and PANCK^+^SPRR3^+^ malignant epithelial cells in the TME. **(K)** Scatter plots showing the correlation between CD8^+^ PD1^+^ T cells and PANCK^+^ SPRR3^+^ malignant epithelial cells in the MUT group (left panel, Pearson’s correlation, R = 0.76) and the WT group (right panel, Pearson’s correlation, R = 0.17).

To elucidate the dynamics of T-cell dysfunction, a developmental trajectory of CD8^+^ T cells was developed using pseudotime analysis. The inferred trajectory revealed a continuous differentiation path originating from progenitor-like cells and culminating in a terminally exhausted state (**Figure 5A**). Sequential and progressive upregulation of exhaustion-related molecules, including PDCD1, LAG3, CTLA4, and TIGIT, was observed along this path, indicating greater dysfunction (**Figures 5B, C**).

Mapping of cells from MUT and WT tumors onto this trajectory revealed marked differences. CD8^+^ T cells from the MUT group were significantly skewed toward the terminally exhausted endpoint, whereas cells from the WT group were more concentrated at earlier stages of the trajectory (**Figures 5D, E**). This indicates that the MUT TME actively promotes the progression of T cells toward irreversible exhaustion.

The extrinsic signals driving this process were then investigated. Analysis of cell-cell communication revealed that interactions originating from the malignant Epi3 and Epi6 subsets were overwhelmingly directed toward T-cell populations, dominating in both interaction number and strength (**Figures 5F, G**). Two predominant signaling axes responsible for this communication were identified in the MUT group: PD-L1 (CD274)-PD-1 and SPP1-CD44. A global visualization of these networks confirmed the central roles of these axes (**Supplementary Figure 2E**). The molecular basis for these interactions was evident in the gene expression patterns, with MUT epithelial cells showing strong expression of the ligands CD274 and SPP1, and exhausted T-cell subsets expressing the corresponding receptors PDCD1 and CD44 (**Supplementary Figure 2F**).

A detailed comparison of these interactions stratified by mutation status confirmed that the signaling strength of specific pairs, particularly SPP1-CD44 and CD274-PDCD1, was markedly stronger in the MUT group (**Supplementary Figure 2G**). Analysis of outgoing and incoming signals further verified that the activities of these two pathways, originating from Epi3 and Epi6, were significantly enhanced in MUT tumors (**Figure 5H**). Furthermore, a strong positive correlation was found between the abundance of the Epi3 subpopulation and the proportions of both CD8_Exhausted (R = 0.79) and IFIT3_T (R = 0.93) cells within the MUT group (**Figure 5I**). As interferon-induced T cell exhaustion is a well-recognized biological phenomenon, this study then focused on Epi3 (30, 31, 36). mIHC staining revealed spatial co-localization between CD8^+^ PD-1^+^ T cells and SPRR3^+^ epithelial cells, with SPRR3 representing a specific marker of the Epi3 subpopulation, suggesting potential interactions between exhausted T cells and this epithelial subset (**Figure 5J**). Correlation analysis indicated that positive associations between CD8^+^ PD-1^+^ T cells and SPRR3^+^ epithelial cells were present only in the MUT group, with no similar association observed in the WT group (**Figure 5K**).

In summary, the analysis indicates a clear mechanistic narrative, in which PIK3CA-mutant epithelial cells, particularly the aggressive Epi3 and inflammatory Epi6 subsets, utilize SPP1 and PD-L1 signaling to actively push CD8^+^ T cells along a differentiation trajectory, ultimately leading to their terminal exhaustion.

### PIK3CA mutations induce a pro-angiogenic niche by expansion of specific macrophage subsets

**Figure 6:**
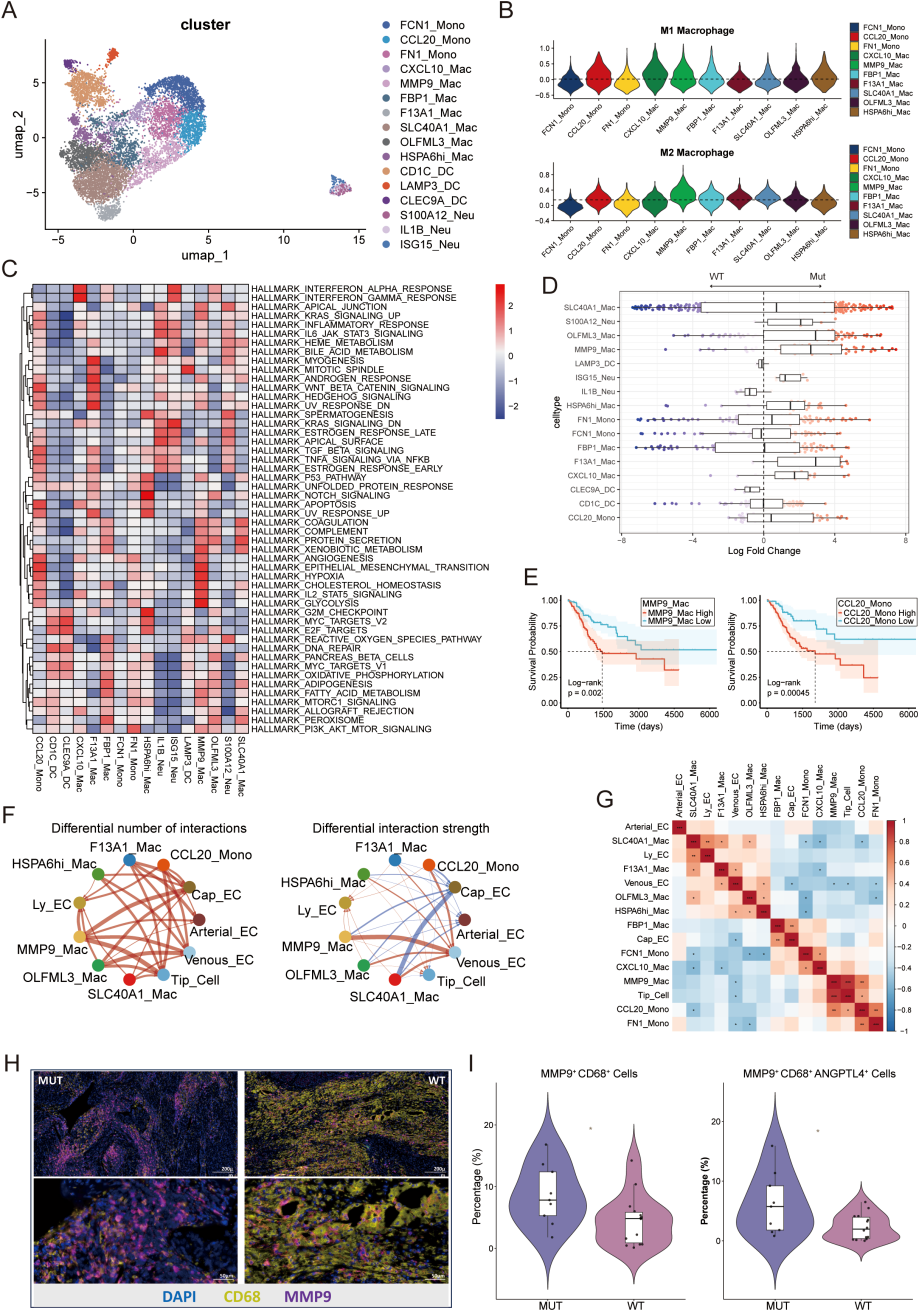
**(A)** UMAP plot showing the reclustering and annotation of myeloid cells into 16 subclusters. **(B)** Violin plots showing the signature scores for M1 Macrophage and M2 Macrophage in all identified myeloid subsets. Scores were calculated using the AddModuleScore function. **(C)** Heatmap showing the hallmark gene signature scores in all identified myeloid subclusters. Scores were calculated using the AddModuleScore function. **(D)** Beeswarm plots overlaid with box plots showing the distribution of neighborhood log2FC values in different myeloid subclusters. Colors correspond to the log2FC as presented in **Figure 2E**. Box plots indicate the median (center line), interquartile range (IQR; box limits), and whiskers extending to 1.5× the IQR. **(E)** Kaplan-Meier survival curves showing the prognostic impact of MMP9_Mac (left) and CCL20_Mac (right) subpopulations, based on Bayesian deconvolution of the TCGA cohort. **(F)** Circle plots illustrating cell-cell communication between myeloid subclusters and endothelial cell subpopulations. The left plot shows the number of interactions; the right plot depicts the interaction strength. **(G)** Heatmap showing Pearson correlations between the relative abundance of myeloid and endothelial subclusters based on single-cell data. Correlation coefficients are indicated by color, and asterisks denote the significance level of the two-sided association test (*p < 0.05, **p < 0.01, ***p < 0.001**). (H)** Representative mIHC staining of MMP9^+^ macrophages in the MUT and WT groups. **(I)** Violin plot showing the proportion of MMP9^+^ macrophages and MMP9^+^ ANGPTL4^+^ macrophages in the MUT and WT groups based on mIHC staining results. Each dot on the violin plot represents an individual sample, *p < 0.05.

We next performed detailed analyses of myeloid and endothelial cells to investigate how PIK3CA mutations remodel the innate immune and stromal compartments. Sixteen distinct myeloid subclusters were identified (**Figure 6A**; **Supplementary Figures 3A, B**). To evaluate the functional phenotypes of these macrophage populations, we first assessed their M1 (classical activation) and M2 (alternative activation) activation states. This scoring revealed that the MMP9_Mac subset exhibited a pronounced tendency toward M2 polarization (**Figure 6B**). Consistent with the tissue-remodeling and immunosuppressive nature of M2 macrophages, further functional annotation indicated that the MMP9_Mac subset was strongly enriched in hypoxia and angiogenesis signatures, while it and the F13A1_Mac subset showed high levels of TGF-β signaling (**Figure 6C**; **Supplementary Figure 3C**). Analysis of differential abundance showed that the MUT TME was significantly enriched with these pro-tumorigenic macrophage populations, most notably MMP9_Mac and F13A1_Mac, along with the monocyte-like CCL20_Mono subset (**Figure 6D**; **Supplementary Figure 3D**). The clinical relevance of these findings was confirmed by survival analyses, where higher abundance of both MMP9_Mac and CCL20_Mono signatures was correlated with significantly poorer patient prognosis (**Figure 6E**).

Given the strong pro-angiogenic signature of these MUT-enriched macrophages, their interactions with the tumor vasculature were then investigated. This identified five functionally distinct endothelial cell (EC) subsets, including the critical Tip_EC subset that initiates vessel sprouting (**Supplementary Figures 3E–G**). Analysis of cell-cell communication revealed robust signaling from the expanded MMP9_Mac and CCL20_Mono populations toward capillary and tip ECs in the MUT group, dominating in both interaction number and strength (**Figure 6F**).

Mechanistically, this communication was mediated primarily by the VEGF and ANGPTL signaling pathways. Analysis of outgoing and incoming signals confirmed significant enhancement of both pathways in the MUT group (**Supplementary Figures 4A, B**). A detailed heatmap of ligand-receptor interactions indicated that specific pairs, such as ANGPTL4–SDC3 and ANGPTL4–CDH5, were the key drivers of this pro-angiogenic crosstalk (**Supplementary Figure 4C**). Correlation analysis provided quantitative evidence for this axis, confirming a significant positive association between the abundance of MMP9_Mac and CCL20_Mono and that of the Tip_EC subset (**Figure 6G**).

The results of mIHC staining further confirmed the enrichment of MMP9^+^ macrophages in the MUT group, indicating their potential involvement in the TME (**Figure 6H**). Moreover, the expression of ANGPTL4 was strongly correlated with MMP9^+^ macrophages, and cells expressing ANGPTL4 were more abundant in the MUT group (**Supplementary Figures 4D, E**). Additionally, MMP9^+^ ANGPTL4^+^ macrophages were also observed to be significantly more enriched in the MUT group, particularly in proximity to blood vessels (**Figure 6I**, **Supplementary Figure S4F**). This confirms that the enrichment of pro-angiogenic macrophages and their signaling to tip cells leads to increased tumor vascularization. Collectively, these findings identify a key mechanism of tumor support in MUT tumors, in which a pro-angiogenic niche functions in concert with direct T-cell inhibition to accelerate tumor progression.

## Discussion

The integration of immunotherapy into the clinical management of advanced CC has been transformative. Nevertheless, effective biomarkers, apart from PD-L1 expression, are urgently needed to optimize patient stratification for ICB treatment. This study investigated the mechanistic role of PIK3CA, the most frequently mutated gene in CC (37), to elucidate the biological basis for its paradoxical, treatment-dependent prognostic significance. The results of the single-cell transcriptomic analysis indicated a mechanistic framework in which PIK3CA mutations promoted a dichotomous TME, characterized by the coexistence of immune inflammation and marked adaptive resistance, thereby creating specific vulnerabilities amenable to combination immunotherapy.

A central finding of the study was the functional specialization of malignant epithelial cells within PIK3CA-mutant tumors. Two distinct subpopulations, Epi3 and Epi6, that showed complementary roles in tumor progression were identified. The Epi3 subset, driven by a BACH1/TEAD1 regulon, exhibited an aggressive, invasive phenotype associated with TGF-β signaling and promotion of the EMT. Concurrently, the Epi6 subset, governed by an IRF9/STAT2 regulon, induced an interferon-rich inflammatory milieu. This functional divergence represents a sophisticated oncogenic strategy. The IFN signature associated with Epi6 can promote a T-cell-inflamed TME, a prerequisite for ICB response. However, this same inflammatory state, coupled with direct signaling from both epithelial subsets, can also drive adaptive immune resistance by inducing terminal exhaustion in the responding T cells (36). This coexistence of immune activation and exhaustion reflects a classic negative feedback loop, where chronic inflammatory signaling inevitably upregulates immune checkpoints. In this context, the proportion of pre-exhausted CD8^+^ T (Tpex) is of critical importance. Tpex cells possess stem-like self-renewal capabilities and are recognized as the primary responders to PD-1 blockade, proliferating and differentiating to exert anti-tumor efficacy. The PIK3CA-mutant TME, rich in both inflammatory signals and pre-exhausted populations, provides the necessary cellular substrate for ICB. The results of the trajectory analysis substantiated this, suggesting that CD8^+^ T cells in mutant tumors were preferentially directed toward irreversible exhaustion. This process was hypothesized to be mediated by epithelial-derived PD-L1 and, notably, SPP1. The identification of the SPP1–CD44 axis as a putative mediator of T-cell dysfunction is a key finding, consistent with recent evidence implicating osteopontin (SPP1) as a pivotal regulator of immunosuppression (38, 39). These dual mechanisms likely underlie the enhanced responsiveness to combined PD-1 inhibitors and anti-angiogenic therapies observed in the clinical cohort, as they address both immune and vascular resistance pathways concurrently.

The results of the study are consistent with those of recent high-impact studies on PI3K pathway alterations in solid tumors, such as those demonstrating the association of PIK3CA mutations with interferon-enriched TMEs in breast cancer (19, 40). However, the present findings diverge from the identification of a dominant exhausted T-cell phenotype in CC rather than the cytotoxic profiles reported in colorectal cancer and other solid tumors (41–43). This difference may stem from the HPV-oncogenesis specific to CC, contrasting with the hormone-dependent context of breast cancer. The novel SPP1–CD44 axis identified as a driver of T-cell exhaustion complements the prevailing focus on PD-L1 in cervical cancer research by elucidating a key non-canonical signaling mechanism (44, 45). Similarly, while the involvement of pro-angiogenic macrophages has been reported in many cancers (46–48), the single-cell resolution in the present study specifically revealed that in PIK3CA-mutated CC, ANGPTL4 secreted by macrophages participates in vascular remodeling, which may represent a potential novel therapeutic target.

The study has a number of important clinical implications. The elucidation of how PIK3CA mutations shape a dual TME landscape enhances the theoretical framework of tumor-immune interactions and provides a mechanistic rationale for the treatment-dependent prognostic paradox of PIK3CA mutations in CC. Clinically, the identification of PIK3CA mutations as predictive biomarkers for combined immunotherapy and anti-angiogenic therapy could guide patient stratification, optimizing therapeutic outcomes for advanced CC. The elucidation of novel signaling axes, such as SPP1-CD44, provides directions for the development of targeted interventions to reverse T-cell exhaustion beyond current ICI treatment. Finally, the integration of the single-cell insights with longitudinal clinical data could refine predictive models for immunotherapy response, while exploration of the metabolic dependencies of PIK3CA-mutant tumors, such as lipid reprogramming, may reveal additional therapeutic vulnerabilities.

This study builds upon genomic and clinical outcome data from two previous clinical studies, one from our team and another from a collaborating group. Nevertheless, there are several limitations. First, due to logistical constraints and the retrospective nature of the sample acquisition, it was not possible to obtain the original tissue specimens from these foundational clinical cohorts for direct verification. Second, our conclusions regarding intercellular interactions, such as the SPP1-CD44 axis driving CD8+ T cell exhaustion, are derived from correlative bioinformatic network inferences and represent mechanistic hypotheses that require future functional validation through *in vitro* and *in vivo* models. Third, the sample size for PIK3CA-mutated tumors in our single-cell dataset is relatively limited. The lack of specific mutation locus annotations in the public datasets precluded a stratified analysis of mutation subtypes, which could introduce unmeasured heterogeneity. Furthermore, all patients included in the analyzed cohorts were HPV-positive. While this reflects the epidemiological reality of cervical cancer, the absence of HPV-negative cases prevented us from exploring the specific synergistic effects between varying HPV infection statuses and PIK3CA mutations. Fourth, the analysis lacks longitudinally collected, pre- and post-treatment paired samples, which would be essential for a direct assessment of the therapeutic modulation of TME. To mitigate these constraints, a comprehensive single-cell dataset of sufficient scale was assembled to ensure analytical rigor. In parallel, mIHC was performed on a separate, independent patient cohort. This experimental validation confirmed the distinct inflammatory and vascular features of the PIK3CA-mutant TME, thereby reinforcing the validity of our conclusions.

In conclusion, the study findings deconstruct the complex TME of PIK3CA-mutant CC, revealing a landscape of co-dependent vulnerabilities. These tumors demonstrated an interplay between T-cell exhaustion and robust angiogenesis, providing a mechanistic explanation for the efficacy of combining ICB with anti-angiogenic therapy. These findings indicate the potential of PIK3CA mutation status as a predictive biomarker to guide precision immunotherapy and suggest promising avenues for translational research for the development of next-generation therapeutic strategies for CC.

## Methods

### scRNA-seq library preparation and data processing

This study integrated single-cell transcriptome data from 21 patients. These included three newly acquired samples (P11, P12, P18) from treatment-naïve patients at Fujian Cancer Hospital, collected under a protocol approved by the hospital’s Institutional Review Board (IRB) with written informed consent from each patient, 18 previously published datasets from Fan et al. (n = 15) (27)and Liu et al. (n = 3) (26). Information on PIK3CA mutations in the Liu cohort was obtained through direct correspondence, while whole-exome sequencing data were available for the Sun cohort. The complete clinical characteristics are summarized in **Supplementary Table 1**.

For the newly collected specimens, a comprehensive workflow was established for the single-cell analysis. Initially, the fresh tumor tissues underwent enzymatic dissociation to generate single-cell suspensions, which were subsequently fixed using the Chromium Next GEM Single Cell Fixed RNA Sample Preparation Kit (PN-1000414) preservation buffer at 4 °C for 16–24 hours. The preserved cell populations were then incubated overnight with specific 10x Genomics probe sets for RNA-probe hybridization. Following hybridization, the samples were labeled with distinct probe barcodes and were combined at equivalent cellular concentrations. After the removal of excess probes, the mixture was processed using the Chromium Next GEM Chip Q Single Cell Kit to generate Gel Beads in EMulsion (GEMs). The resulting products underwent an eight-cycle pre-amplification step, followed by sequential adapter attachment and size selection for preparation of the final sequencing libraries, which were subsequently analyzed on the Illumina NovaSeq platform (Illumina, San Diego, CA, USA) using paired-end 150-bp sequencing.

The raw sequencing data were processed using the CellRanger pipeline to generate expression matrices. Quality control measures were implemented across all samples, including those obtained from public repositories. Potential doublets were eliminated, together with the application of stringent filtering criteria, retaining cells that expressed more than 200 genes, contained over 1,000 unique molecular identifiers (UMIs), and showed less than 15% mitochondrial gene content. The filtered data underwent library-size normalization and log transformation using Seurat, generating a normalized expression matrix for subsequent analyses.

### Subpopulation clustering and cell type annotation

The “RunHarmony” function for data integration to correct for batch effects, consistently using the top 30 principal components (PCs) in all subsequent analyses. Following integration, the expression matrix was normalized with the “NormalizeData” function in Seurat, and highly variable genes (HVGs) were identified using “FindVariableFeatures.” The data were then scaled with “ScaleData,” and dimensionality reduction was performed using “RunPCA” based on the first 30 PCs. Clustering was conducted by the construction of a shared nearest-neighbor graph with “FindNeighbors” and the identification of clusters with “FindClusters,” both using the first 30 PCs, and the results were visualized using Uniform Manifold Approximation and Projection (UMAP). Marker genes for each cluster were identified with the “FindAllMarkers” function, and cell type annotation was performed based on documented marker genes.

### CNV estimation in different epithelial subpopulations

Somatic copy number variations (CNVs) at the single-cell resolution were identified using epithelial cells from uterine leiomyoma as a reference population. CNV profiling was conducted using the R package inferCNV (version 1.20.0), enabling the detection of large-scale chromosomal alterations based on transcriptomic data. CNV scores were calculated for each cell, defined as the mean of the squared CNV values within the genome, to quantitatively assess the degree of genomic instability. This scoring metric facilitated the systematic evaluation of the malignant potential of epithelial cells. The CNV patterns within the individual epithelial subclusters were visualized to assess the heterogeneity of genomic alterations and characterize differences in malignant propensity in different subpopulations.

### Pathway enrichment and gene set scoring

The biological functions and associated signaling pathways were compared between the MUT and WT groups, as well as different cell clusters, using Gene Ontology (GO) and Kyoto Encyclopedia of Genes and Genomes (KEGG) enrichment analyses with the clusterProfiler R package. Differentially expressed genes (DEGs) for each cluster or group were annotated and mapped to Entrez IDs, and enrichment analyses were conducted for both GO annotations and KEGG pathways.

In addition, Hallmark gene set scores were calculated for each cell based on the MSigDB Hallmark collection, and differences between clusters or groups were assessed using linear modeling with the limma package in R. To further characterize immune cell functional states, gene sets defining T cell functions (such as cytotoxic, exhausted, and costimulatory signatures) and macrophage functions were identified from published studies (49, 50). Cell function scores based on these specific gene sets were calculated using the built-in AddModuleScore function in the Seurat package.

### Analysis of metabolic pathway activities

To explore the metabolic characteristics of different epithelial subpopulations, metabolic pathway activity was evaluated at the single-cell level using the scMetabolism package. Expression data were processed to infer the activities of KEGG-defined metabolic pathways identified for each cell, and differential metabolic activity among the epithelial subpopulations was identified using linear modeling.

### Cell-cell interaction analysis

Intercellular crosstalk was evaluated by analysis of the communication networks between epithelial, myeloid, and T-cell populations using the R package **CellChat**. The analysis was conducted separately on the WT and MUT datasets to identify condition-specific interactions. The two resulting CellChat objects were merged to determine the differential interaction strengths and signaling pathways between the WT and MUT conditions. Key dysregulated pathways and their constituent ligand-receptor pairs were then visualized to illustrate changes in cell communication patterns within the TME.

### Analysis of differential abundance in the scRNA-seq data

Differential abundance was assessed to investigate changes in the composition of cell populations. For major cell lineages, overall shifts were examined in the healthy donor (HD), WT, and MUT cohorts by calculating odds ratios (OR) to quantify the relative enrichment of each cell type between conditions. For a higher-resolution analysis within the CD8^+^ T cell and myeloid compartments, MiloR was used to detect fine-grained shifts in cell state abundance. A k-nearest neighbor (KNN) graph was constructed from the integrated data using the buildGraph function, with k = 20 and based on the top 30 principal components. Neighborhoods within the graph were subsequently defined using the makeNhoods function. To enhance computational performance, the analysis was performed using a randomly selected 20% subset of the cells. Differential abundance was tested by fitting a negative binomial generalized linear model (NB-GLM) to the cell counts within each neighborhood, with the application of TMM normalization to adjust for differences in library size among the samples. The resulting neighborhood-level log-fold changes between conditions were then visualized to identify cellular states exhibiting significant alterations.

### Inference of gene regulatory networks

The regulatory gene networks controlling epithelial cell states were assessed by Single-Cell Regulatory Network Inference and Clustering (SCENIC) analysis using the R package implementation. The analysis comprised three main stages. First, co-expression modules were inferred between transcription factors (TFs) and potential target genes from the epithelial cell raw count matrix using GENIE3. Second, these modules were refined using cis-regulatory motif analysis with RcisTarget, retaining only TF-target modules with significant motif enrichment for the corresponding TF. A TF and its validated direct targets represented a “regulon”. In the final step, the activity of each regulon within every cell was quantified using AUCell, which calculated the area under the recovery curve, yielding a regulon activity matrix.

### Regulon module analysis

Co-regulated groups of regulons were identified using a module analysis based on their activity profiles. The Connection Specificity Index (CSI) was first calculated for all regulon pairs; this measures the specificity of their correlations. A CSI matrix was generated, followed by hierarchical clustering using the Euclidean distance to group regulons into distinct modules. This process identified eight core regulon modules operating within the epithelial compartment.

### Bulk tissue deconvolution using BayesPrism

The fractional abundance of the defined single-cell populations within bulk tumor samples was estimated by digital cytometry using BayesPrism. A comprehensive single-cell reference profile was constructed using all annotated cell subtypes from the dataset. The raw count matrix from the single-cell data served as the reference input.

Before deconvolution, rigorous gene filtering was performed on the reference profile to remove genes known to introduce technical bias. The reference was further refined to include only protein-coding genes. This filtered single-cell reference profile, along with bulk RNA-seq count data from TCGA, was used to construct a prism object. The Bayesian deconvolution algorithm was then used to infer the cell-type proportions for each TCGA tumor sample. These estimated cell fractions were then used for survival analysis and assessment of their prognostic significance.

### Immune microenvironment analysis of the TCGA-CESC cohort

Somatic mutation data (MAF) and corresponding RNA-sequencing expression profiles for the Cervical Cancer (CESC) cohort were obtained from TCGA. The expression matrix was normalized to log2-transformed Transcripts Per Million (TPM). Based on the mutation data, patients were stratified into PIK3CA-mutant (MUT) and PIK3CA-WT groups. To characterize the tumor immune microenvironment,ssGSEA was performed using the GSVA R package. Based on previously defined gene sets, the enrichment scores for 28 immune cell populations were calculated. Differences in the abundance of these immune cell types between the PIK3CA-MUT and WT groups were assessed using the Wilcoxon rank-sum test, with a p-value < 0.05 considered statistically significant.

### Multiplexed immunofluorescence staining

Multiplex immunofluorescence staining was performed on formalin-fixed paraffin-embedded (FFPE) tissue sections using the Think 7-plex fluorescent mIHC kit (FreeThinking Biosciences, Nanjing, China) according to the manufacturer’s instructions. Sections were first deparaffinized and rehydrated, followed by heat-induced epitope retrieval (HIER). The staining protocol targeted two separate marker panels: the first included PANCK (Abcam, Cat#ab308262), PD1 (Abcam, Cat#ab237728), SPRR3 (Proteintech, Cat#11742-1-AP), and CD8 (CST, Cat#85336), while the second comprised MMP9 (Abcam, Cat#ab76003), CD31 (Abcam, Cat#ab281583), CD68(CST, Cat#76437), and ANGPTL4 (Abcam, Cat#ab206420). For each staining cycle, sections were incubated with the appropriate anti-human primary antibody, endogenous peroxidase activity was blocked, and a polymeric HRP-conjugated secondary antibody was applied. The signals were then developed using a fluorophore-conjugated Tyramide Signal Amplification (TSA) kit (FreeThinking Biosciences) at a 1:500 dilution. Following each TSA signal deposition, HIER was repeated to remove antibody complexes while preserving the fluorescent signal, and this cycle was repeated until all markers in a panel were labeled. Finally, the slides were counterstained with DAPI to visualize the nuclei. Fluorescence images were acquired using a Vectra Polaris fluorescence scanner (Akoya Bio, MA, USA), and quantitative analysis was performed using the HALO Highplex FL analysis module (v3.4.2986.257, Indica Labs, Albuquerque, NM, USA).

### Digital PCR-based detection of PIK3CA mutations in single-cell samples

A total of 18 tumor samples were included for PIK3CA mutation analysis. Twelve samples were obtained from the supplementary figures of the study by Fan et al. (27), in which mutation status was publicly available. Three additional FFPE tumor specimens were obtained from Liu et al. (51) after direct communication with the authors. These three samples, together with three FFPE tumor samples from our own cohort, were subjected to mutation detection using the method described below.

Formalin-fixed paraffin-embedded (FFPE) tissue specimens were processed for the detection of PIK3CA mutations in exons 7, 9, and 20 by digital PCR (dPCR). Genomic DNA was extracted from FFPE sections using a magnetic bead–based DNA extraction kit (CretBiotech, CNA01790S) according to the manufacturer’s instructions, with modifications to ensure complete paraffin removal. Briefly, deparaffinization was performed at 58 °C with Buffer DS, followed by ethanol washes and proteinase K digestion at 58 °C for 60 min. Samples were subsequently heat-treated at 90 °C for 60 min, subjected to magnetic bead purification, and eluted in Buffer EB.

Digital PCR assays were performed using a commercial PIK3CA mutation detection kit (Xinyi, 12245-1) capable of detecting 11 mutations across the target exons. DNA templates were diluted to 2 ng/μL and combined with assay-specific PCR reagents (Mix-B and Mix-C). Droplets were generated using a droplet generation system with droplet generation oil, then subjected to PCR amplification (95 °C for 10 min; 40 cycles of 94 °C for 30 s and 60 °C for 1 min; final hold at 12 °C for 5 min). Amplified droplets were analyzed using a biochip reader (Xinyi TD-2), and positive droplets were identified based on fluorescence signals. Mutation status was determined in accordance with the manufacturer’s analysis guidelines.

## Data Availability

The data presented in this study are deposited in the Genome Sequence Archive for Human repository at the National Genomics Data Center, China National Center for Bioinformation / Beijing Institute of Genomics, Chinese Academy of Sciences, under accession number HRA016956, and are publicly available at https://ngdc.cncb.ac.cn/gsa-human. Other publicly available sequencing datasets used in this study can be downloaded from the corresponding databases.

## Supplementary material

Supplementary Figure 1 **(A)** Word cloud visualization of genes harboring ≥ 20 somatic mutations in the cohort, with font size scaled to mutation frequency, highlighting those showing the highest frequency of alteration. **(B)** Pairwise interaction matrix illustrating significant patterns of somatic co-occurrence (brown) and mutual exclusivity (green) among the 20 most frequently mutated genes in the merged cohort, with circle size indicating statistical significance and annotated interactions denoting adjusted P-values below threshold. **(C)** Scatter plot from the OncodriveCLUST analysis depicting the Z-score versus the fraction of clustered mutations for each gene, with point size corresponding to the total mutation count and genes exceeding the FDR threshold (0.20) labeled as significant positional drivers. **(D)** Violin plots showing differences in immune infiltration between PIK3CA-MUT and WT CC tumors based on ssGSEA scores from the TCGA RNA-seq data. Statistical significance was assessed using the Wilcoxon rank-sum test; *p < 0.05. **(E)** UMAP plots displaying cells from individual samples to assess batch effects. Each subplot corresponds to a single sample. **(F)** UMAP visualization of batch effects in the different sample groups.

Supplementary Figure 2 **(A)** Heatmap showing the top five signature genes for each NK/T cell subcluster. **(B)** Violin plots showing the expression levels of selected marker genes in the XCL1⁺ NK, FGFBP2⁺ NK, and KIT⁺ NK clusters. **(C)** Differential abundance of the FGFBP2⁺ NK cell subset between the PIK3CA-MUT and WT groups, based on deconvolution of bulk RNA-seq data from the TCGA CC cohort. **(D)** Kaplan-Meier survival analysis comparing overall survival between patients with high vs. low infiltration of FGFBP2⁺ NK cells, based on stratification of the TCGA CC cohort. **(E)** Heatmap comparing ligand–receptor signaling interactions between the Epi3 and Epi6 subclusters and NK/T cell subpopulations, stratified by PIK3CA mutation status. **(F)** Violin plots illustrating the expression of key genes involved in the PD-L1-PD-1 and SPP1-CD44 signaling axes in different cell subpopulations. **(G)** Circular plot displaying the communication network between the SPP1 (top) and PD-L1 (bottom) signaling pathways.

Supplementary Figure 3 **(A)** UMAP feature plots displaying the expression of marker genes by different myeloid subpopulations. **(B)** Heatmap showing the top five signature genes in each myeloid subcluster. **(C)** Heatmap showing the expression of characteristic functional genes in the different myeloid clusters. **(D)** Bar plot showing the proportions of myeloid subpopulations in the UL, MUT, and WT groups. **(E)** UMAP plot showing the subcluster distribution of endothelial cells, together with batch information. **(F)** UMAP feature plots displaying the expression of marker genes for different endothelial subpopulations. **(G)** Heatmap showing the top five signature genes in the different endothelial subclusters.

Supplementary Figure 4 **(A)** Bar plots showing the relative proportion of cell-cell communication signals between myeloid and endothelial subpopulations (left) and the relative signal strength (right). **(B)** Outgoing and incoming signals of VEGF and ANGPTL communication pathways in the MUT and WT groups. **(C)** Heatmap illustrating the differential cell-cell communication strength of VEGF and ANGPTL ligand-receptor pairs between myeloid and endothelial subpopulations in the MUT and WT groups. **(D)** Scatter plots showing correlations between ANGPTL4+ cells and MMP9+ macrophages in the WT group (left panel, Pearson’s correlation, R = 0.73) and the MUT group (right panel, Pearson’s correlation, R = 0.85). **(E)** Violin plot showing the proportion of ANGPTL4+ cells in the MUT and WT groups based on mIHC staining results. Each dot on the violin plot represents an individual sample, *p < 0.05. **(F)** Representative mIHC staining showing the perivascular localization of SPP1⁺ ANGPTL4⁺ macrophages in the TME.

Supplementary Table 1 Sample information.
