# Supplementary figures and images for: PIK3CA mutation–induced immune microenvironment remodeling sensitizes cervical cancer to immunotherapy
Source: Front Immunol. 2026 Mar 25;17:1780752. doi: 10.3389/fimmu.2026.1780752 (PMC13057328; doi:10.3389/fimmu.2026.1780752)

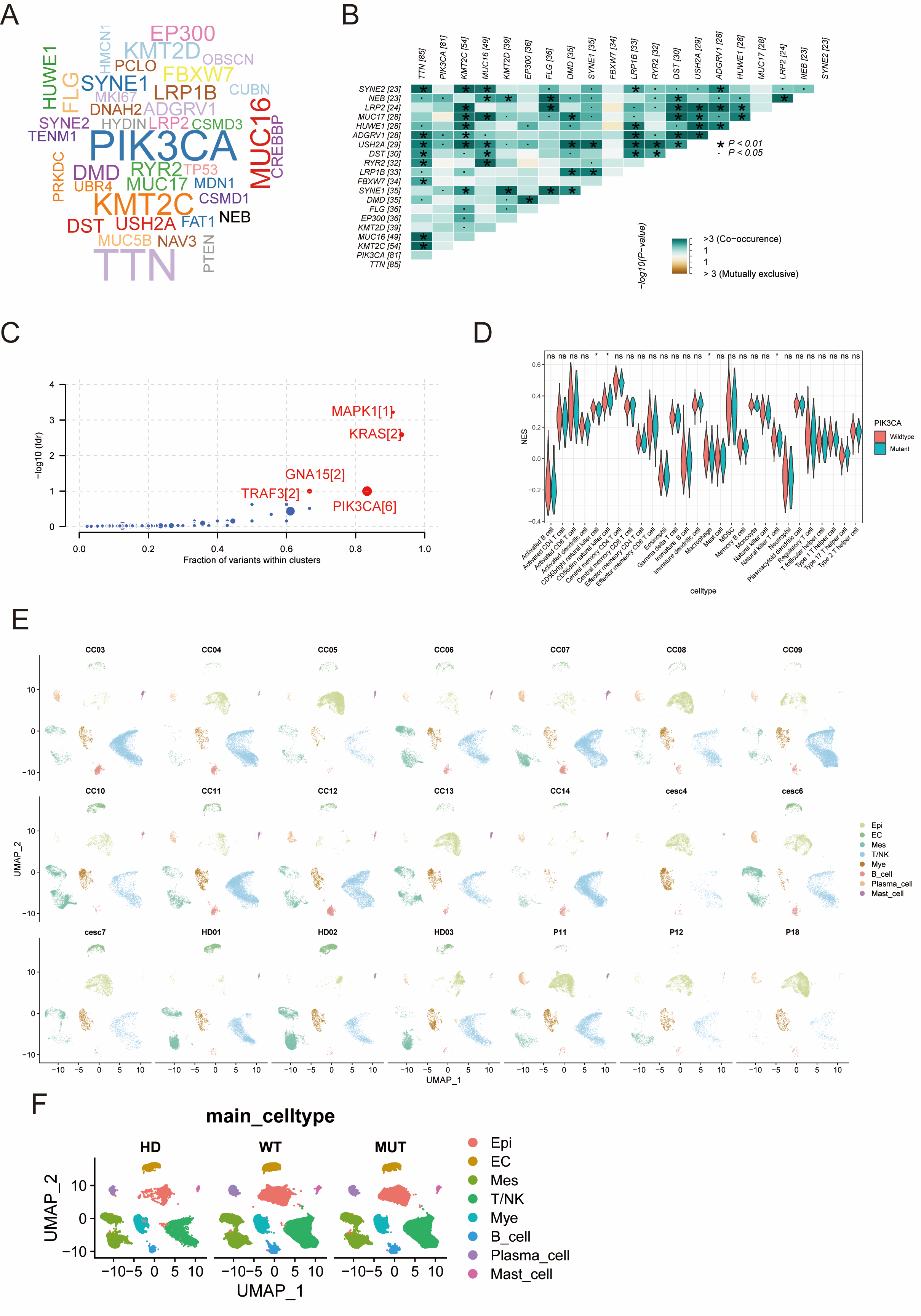

Supplement: Supplementary Figure 1 — (A) Word cloud visualization of genes harboring ≥ 20 somatic mutations in the cohort, with font size scaled to mutation frequency, highlighting those showing the highest frequency of alteration. (B) Pairwise interaction matrix illustrating significant patterns of somatic co-occurrence (brown) and mutual exclusivity (green) among the 20 most frequently mutated genes in the merged cohort, with circle size indicating statistical significance and annotated interactions denoting adjusted P-values below threshold. (C) Scatter plot from the OncodriveCLUST analysis depicting the Z-score versus the fraction of clustered mutations for each gene, with point size corresponding to the total mutation count and genes exceeding the FDR threshold (0.20) labeled as significant positional drivers. (D) Violin plots showing differences in immune infiltration between PIK3CA-MUT and WT CC tumors based on ssGSEA scores from the TCGA RNA-seq data. Statistical significance was assessed using the Wilcoxon rank-sum test; *p < 0.05. (E) UMAP plots displaying cells from individual samples to assess batch effects. Each subplot corresponds to a single sample. (F) UMAP visualization of batch effects in the different sample groups. [file Image1.tif]

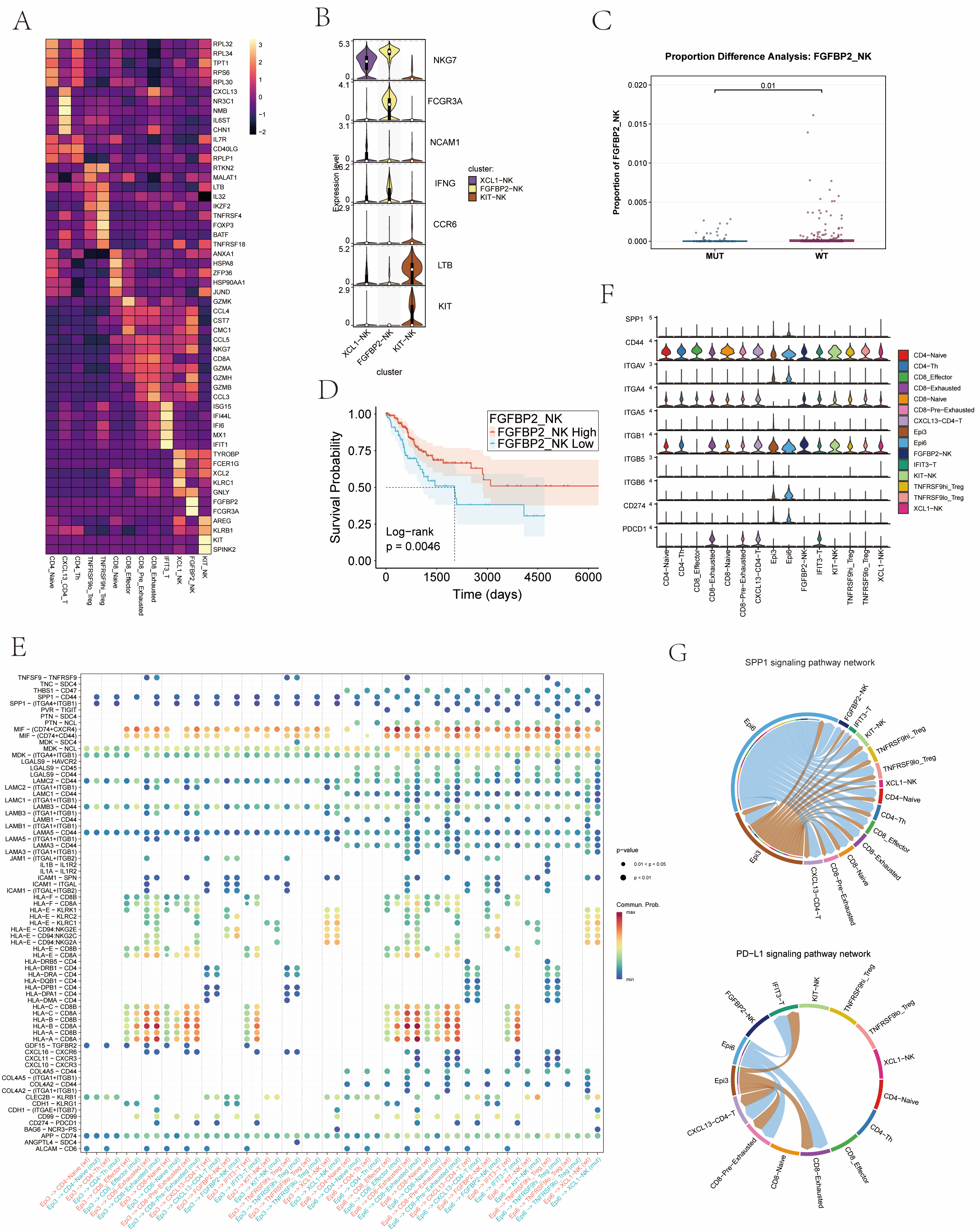

Supplement: Supplementary Figure 2 — (A) Heatmap showing the top five signature genes for each NK/T cell subcluster. (B) Violin plots showing the expression levels of selected marker genes in the XCL1⁺ NK, FGFBP2⁺ NK, and KIT⁺ NK clusters. (C) Differential abundance of the FGFBP2⁺ NK cell subset between the PIK3CA-MUT and WT groups, based on deconvolution of bulk RNA-seq data from the TCGA CC cohort. (D) Kaplan-Meier survival analysis comparing overall survival between patients with high vs. low infiltration of FGFBP2⁺ NK cells, based on stratification of the TCGA CC cohort. (E) Heatmap comparing ligand–receptor signaling interactions between the Epi3 and Epi6 subclusters and NK/T cell subpopulations, stratified by PIK3CA mutation status. (F) Violin plots illustrating the expression of key genes involved in the PD-L1-PD-1 and SPP1-CD44 signaling axes in different cell subpopulations. (G) Circular plot displaying the communication network between the SPP1 (top) and PD-L1 (bottom) signaling pathways. [file Image2.tif]

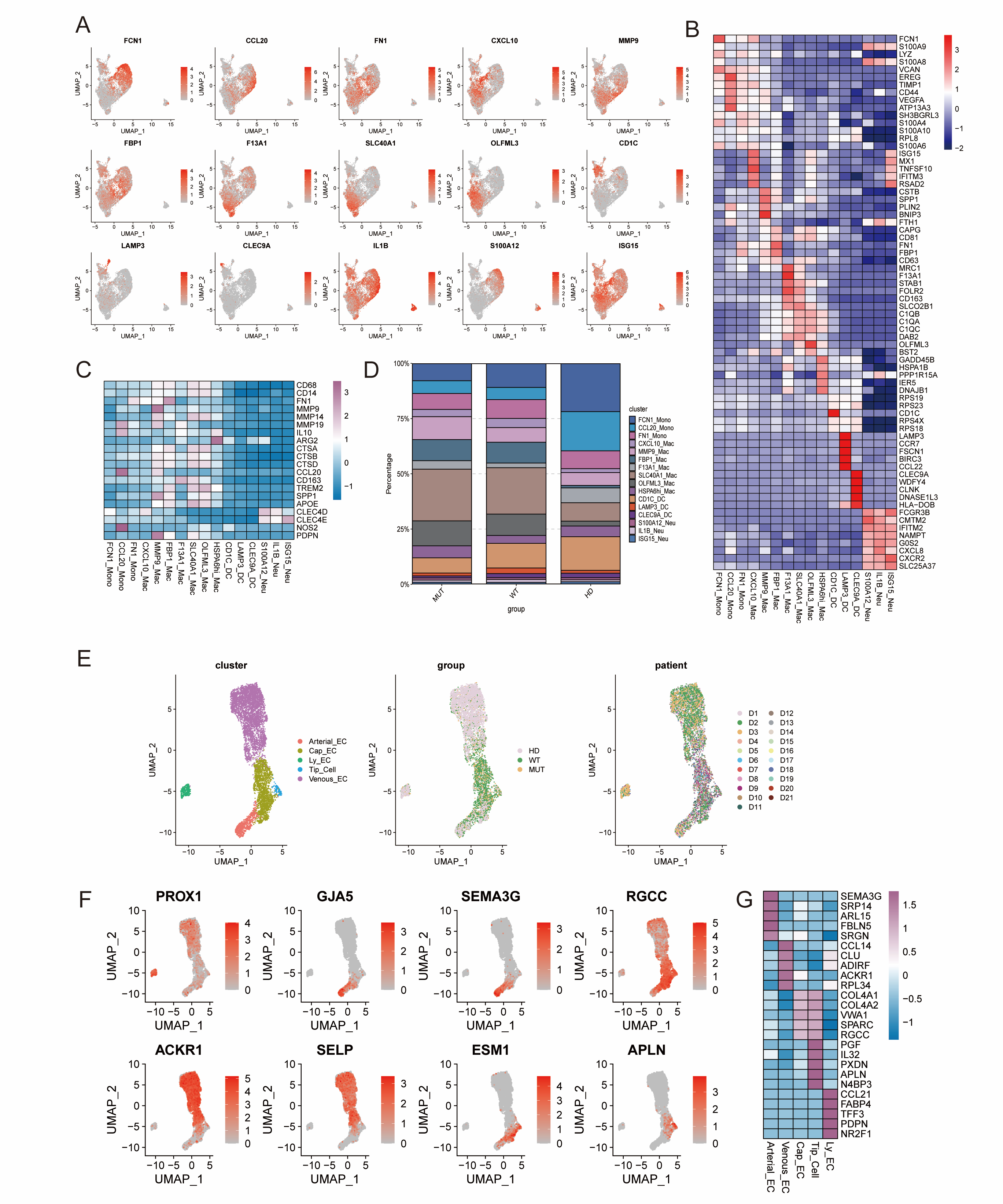

Supplement: Supplementary Figure 3 — (A) UMAP feature plots displaying the expression of marker genes by different myeloid subpopulations. (B) Heatmap showing the top five signature genes in each myeloid subcluster. (C) Heatmap showing the expression of characteristic functional genes in the different myeloid clusters. (D) Bar plot showing the proportions of myeloid subpopulations in the UL, MUT, and WT groups. (E) UMAP plot showing the subcluster distribution of endothelial cells, together with batch information. (F) UMAP feature plots displaying the expression of marker genes for different endothelial subpopulations. (G) Heatmap showing the top five signature genes in the different endothelial subclusters. [file Image3.tif]

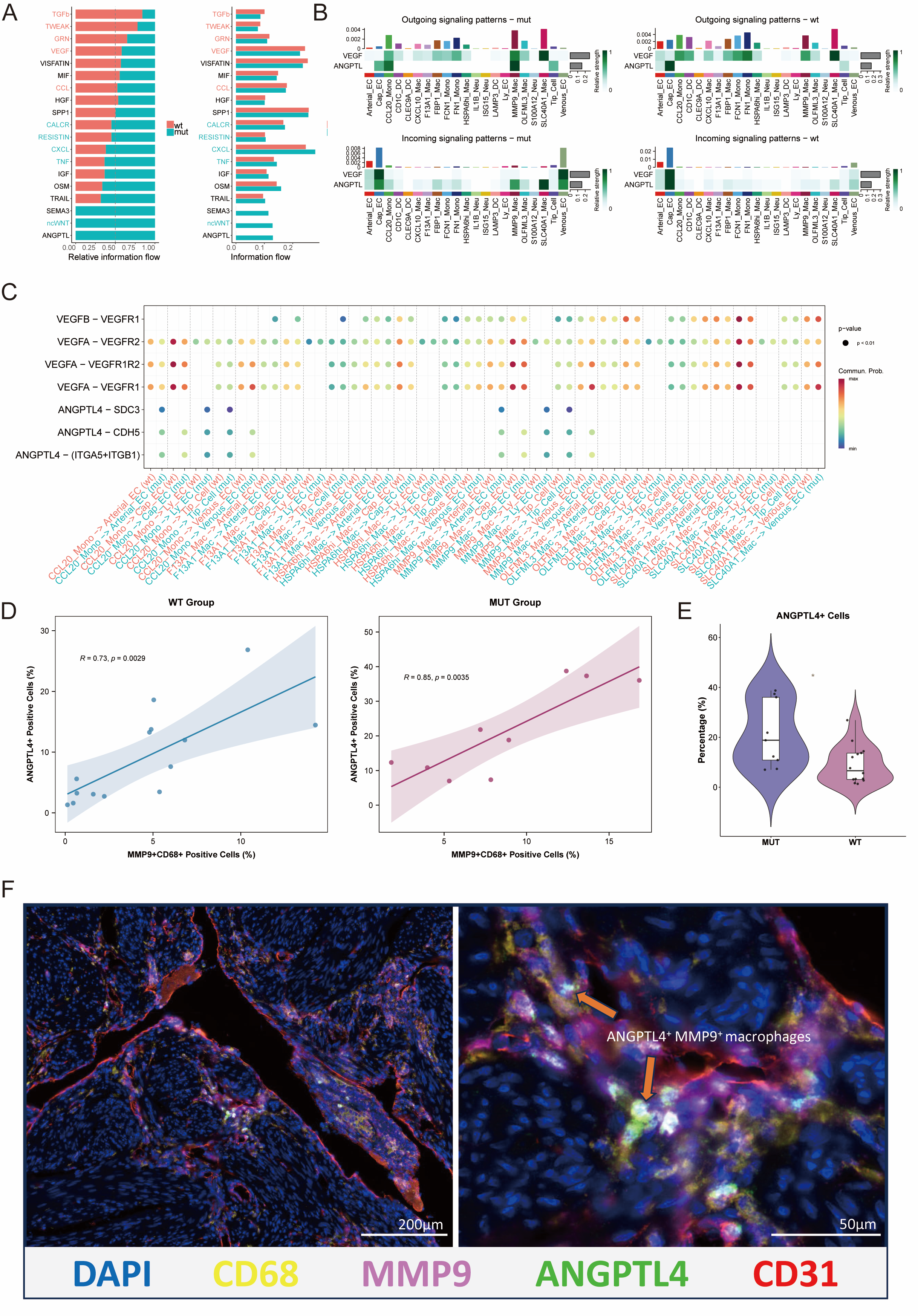

Supplement: Supplementary Figure 4 — (A) Bar plots showing the relative proportion of cell-cell communication signals between myeloid and endothelial subpopulations (left) and the relative signal strength (right). (B) Outgoing and incoming signals of VEGF and ANGPTL communication pathways in the MUT and WT groups. (C) Heatmap illustrating the differential cell-cell communication strength of VEGF and ANGPTL ligand-receptor pairs between myeloid and endothelial subpopulations in the MUT and WT groups. (D) Scatter plots showing correlations between ANGPTL4+ cells and MMP9+ macrophages in the WT group (left panel, Pearson’s correlation, R = 0.73) and the MUT group (right panel, Pearson’s correlation, R = 0.85). (E) Violin plot showing the proportion of ANGPTL4+ cells in the MUT and WT groups based on mIHC staining results. Each dot on the violin plot represents an individual sample, *p < 0.05. (F) Representative mIHC staining showing the perivascular localization of SPP1⁺ ANGPTL4⁺ macrophages in the TME. [file Image4.tif]
